# Dissect the immunity using cytokine profiling and NF-kB target gene analysis in systemic inflammatory minipig model

**DOI:** 10.1371/journal.pone.0252947

**Published:** 2021-06-04

**Authors:** Han Na Suh, Young Kyu Kim, Ju Young Lee, Goo-Hwa Kang, Jeong Ho Hwang

**Affiliations:** Animal Model Research Group, Korea Institute of Toxicology, Jeongeup, Jeollabuk-do, Republic of Korea; University of Michigan Health System, UNITED STATES

## Abstract

Minipigs have remarkably similar physiology to humans, therefore, they it can be a good animal model for inflammation study. Thus, the conventional (serum chemistry, histopathology) and novel analytic tools [immune cell identification in tissue, cytokine level in peripheral blood mononuclear cells (PBMC) and serum, NF-kB target gene analysis in tissue] were applied to determine inflammation in Chicago Miniature Swine (CMS) minipig. Lipopolysaccharide (LPS)-induced acute systemic inflammation caused liver and kidney damage in serum chemistry and histopathology. Immunohistochemistry (IHC) also showed an increase of immune cell distribution in spleen and lung during inflammation. Moreover, NF-kB-target gene expression was upregulated in lung and kidney in acute inflammation and in heart, liver, and intestine in chronic inflammation. Cytokine mRNA was elevated in PBMC under acute inflammation along with elevated absolute cytokine levels in serum. Overall, LPS-mediated systemic inflammation affects the various organs, and can be detected by IHC of immune cells, gene analysis in PBMC, and measuring the absolute cytokine in serum along with conventional inflammation analytic tools.

## Introduction

Inflammation is a well-organized response of immune cells and biomolecules. When the body undergoes inflammation, it initiates eicosanoids, chemokines, and cytokines released by resident macrophages or mast cells, and then recruits neutrophils and lymphocytes. These combined responses induce typical inflammatory symptoms and pathophysiological conditions. Inflammation is classified as either acute or chronic based on duration or cause. Acute inflammation is rapid response initiated by infection or tissue damage which follows abnormal vascular permeability, blood flow, and nerve fiber sensitization [1]. Chronic inflammation is long-term response caused by repeated tissue injury and recovery [2] is closely linked with a number of diseases (ischemic disease, atherosclerosis, stroke, cancer, diabetes mellitus, non-alcoholic fatty liver disease) [3].

Inflammation animal models have been established to find new drugs and explore the pathophysiological mechanism of inflammation. To mimic a local inflammation (chemical-induced paw or ear edema animal model) [4] or systemic inflammation (LPS administration, *Escherichia coli* inoculation, or cecal ligation and puncture), various methods are utilized [5]. Among them, LPS is a potent immune stimulator through CD14/toll-like receptor 4 (TLR4)/myeloid differentiation 2 (MD2) receptor in monocyte/macrophage. As LPS-induced cytokines mediate loss of function and immune cell infiltration in tissues, intravenous LPS provokes sepsis-like systemic inflammation [6]. Local LPS administration generates respiratory [7] or neural inflammation [8, 9]. LPS-challenged animal models show a rapid onset of inflammation and similar symptoms to humans. In addition, LPS affects both immune cells and parenchymal cells, including alveolar epithelial cells, myocardial cells, and kidney tubule cells [10–13], suggesting that LPS directly and indirectly modulates the immune response in various organs. Therefore, in this study, LPS was chosen to induce systemic inflammation in minipig model.

So far, rodents have been widely used as an inflammation animal model because cost effectiveness and easy of handling [14]. However, for LPS, a dose 10^6^ times greater is required to induce similar symptoms in mice as in humans. Non-human primates (NHPs) are also used as inflammatory animal models due to their phylogenic proximity to humans. NHPs are more resistant to LPS than human [15, 16], but display an identical hemodynamic and cytokine response under LPS [15]. There is evidence that strong LPS resistance in mice is related with protein factors in rodent sera, which are absent in humans [17]. Immune system similarity may provide a better inflammatory animal model. Immune gene analysis revealed that porcine shows higher similarity, at DNA sequence level, to humans than mice [18]. In particular, porcine immune response shows 80% similarity as human than 10% similarity of mice [19]. Thus, the minipig was chosen as a systemic inflammatory animal model. In this study, the conventional diagnostic tools (serum chemistry, histopathology) are compared with direct immune cell detection (IHC) and LPS-induced gene expression in CMS minipig systemic inflammation model.

## Materials and methods

### Experimental animals

Six minipigs, (CMS, *Sus Scrofa*), 10 months-old, 19.60–24.65 kg body weight, were used. Two minipigs were randomly selected, based on body weight for each group. To establish the systemic inflammatory minipig model, 5 μg/kg LPS was administered seven times intramuscularly for chronic inflammation, and 25 μg/kg LPS was administered once intramuscularly for acute inflammation. Minipigs were housed under a 12 h/12 h light/dark cycle with lights on at 8 am. Water was provided *ad libitum* and food was provided at 2% of body weight per day. All the animal experiments were conducted under the Institutional Animal Care and Use Committee guideline of Korea Institute of Toxicology (IACUC approval # 19-1-0194, 20-1-0064).

### Serum chemistry

For serum chemistry, blood was collected, incubated for 30 min at RT, and centrifuged for 10 min, 3,000 rpm at RT. Supernatant was isolated as serum. Serum chemistry was measured using TBA 120 FR chemistry analyzer (Toshiba Co., Japan).

### Histological analysis

#### Histopathology

Tissues from target organs (kidney, liver) were fixed in 10% neutral buffered formalin (NBF) overnight and embedded in paraffin. Tissue samples were sectioned (5 μm), deparaffinized, and stained with hematoxylin and eosin (H&E) to determine structural abnormalities of kidney and liver. Glomeruli (black dot line) and proximal tubules were examined in kidney. Hepatic tissues near central vein (CV, black dot line) were examined in liver.

#### Immunohistochemistry

Tissues were fixed in 10% NBF overnight and embedded in paraffin. Tissue samples were then sectioned (5 μm), deparaffinized, processed for antigen retrieval, blocked, incubated with target primary antibody, and peroxidase-conjugated secondary antibody. Samples were mounted and photographed using microscopy (Leica DM2700). For peroxidase-conjugated secondary antibody, 3,3’-Diaminobenzidine (DAB) substrate was used, followed by hematoxylin for nuclear counterstaining.

### Gene analysis

For gene expression analysis, tissues (heart, lung, kidney, liver, duodenum, PBMC) were processed for RNA extraction (QIAGEN RNeasy Mini Kit) and reverse transcription (iScript RT Supermix for RT-qPCR, Biorad). Glyceraldehyde-3-Phosphate Dehydrogenase (GAPDH) was used as an endogenous control for normalization. qRT-PCR was performed using intron-spanning primers. Fold induction was quantified using the 2^−ΔΔCT^ method. In Fig 4A, values are displayed using Heatmap software (bar.utoronto.ca/). Primer sequences are listed on Table 1.

**Table 1:** Primers used for qRT-PCR.

Species	Gene symbol	Primer sequences (from 5’ to 3’)	Length	Gene Bank ID
	*CRP*	F: AGGGCGCTGAGGTATGAAAT	117	NM_213844.2
		R: ACAAGGGGAACGTAAGGTGT		
	*SOD1*	F: AGGCCGTGTGTGTGCTGAA	117	NM_001190422.1
		R: GATCACCTTCAGCCAGTCCTTTA		
	*IL-1β*	F: GAGCATCAGGCAGATGGTGT	134	NM_214055.1
		R: CAAGGATGATGGGCTCTTCTTC		
	*IL-6*	F: GCTGCTTCTGGTGATGGCTACTGCC	318	NM_001252429.1
		R: TGAAACTCCACAAGACCGGTGGTGA		
	*TNFα*	F: ATGAGCACTGAGAGCATGATCCG	163	NM_214022.1
*Sus scrofa*		R: CCTCGAAGTGCAGTAGGCAGA		
	*COX2*	F: TTCAACCAGCAATTCCAATACCA	87	NM_214321.1
		R: GAAGGCGTCAGGCAGAAG		
	*TGFβ*	F: AGGGCTACCATGCCAATTTCT	101	NM_214015.2
		R: CGGGTTGTGCTGGTTGTACA		
	*IL-10*	F: CGG CGC TGT CAT CAA TTT CTG	89	NM_214041.1
		R: CCC CTC TCT TGG AGC TTG CTA		
	*GAPDH*	F: ACAGACAGCCGTGTGTTCC	62	NM_001206359.1
		R: ACCTTCACCATCGTGTCTCA		

### Enzyme-Linked Immunosorbent Assay (ELISA)

To measure the absolute cytokine level, serum was obtained at day 0 (pre-treatment), day 1~day7 (two hours after LPS administration], and day 8 (post-treatment). Porcine IL-1β (R&D, PLB00B), IL-6 (R&D, P6000B), TNFα (R&D, TPA00), IL-8 (Invitrogen, P8000), and IFNγ (R&D, DY985) were measured by the ELISA method according to the manufacturer’s protocol. The absolute cytokine levels are shown in Table 2.

**Table 2:** Absolute cytokine level after LPS induction.

	IL-1β (pg/ml)		IL-6 (pg/ml)		TNFα (pg/ml)	
	Chronic	Acute	Chronic	Acute	Chronic	Acute
**D0**	U.D.	U.D.	0.80 ± 0.51	5.02 ± 1.53	27.78 ± 1.50	88.21 ± 2.51
**D1**	39.03 ± 4.3	5.72 ± 1.16	977.5 ± 10.27	6.58 ± 0.37	2312 ± 49.54	74.74 ± 2.77
**D2**	135 ± 9.13	15.37 ± 8.61	80.89 ± 2.92	7.44 ± 0.60	749.9 ± 9.77	63.43 ± 2.15
**D3**	112.6 ± 10.73	5.036 ± 0.20	45.47 ± 1.42	5.1 ± 0.68	442.7 ± 4.09	75.46 ± 1.08
**D4**	40.57 ± 1.36	14.06 ± 6.85	12.43 ± 0.75	6.65 ± 1.27	210.6 ± 4.92	74.75 ± 1.24
**D5**	86.69 ± 10.52	11.38 ± 4.93	11.54 ± 2.66	6.26 ± 0.11	108.8 ± 3.89	81.88 ± 1.06
**D6**	23.69 ± 13.95	32.23± 16.89	7.06 ± 0.48	7.92 ± 1.17	114 ± 2.57	77.19 ± 2.15
**D7**	63.62 ± 7.58	716.3 ± 6.21	15.99 ± 1.09	2218 ± 122.3	111.4 ± 0.95	9224 ± 1218
**D8**	21.37 ± 6.14	96.22 ± 5.23	2.35 ± 0.46	30.05 ± 0.64	25.72 ± 1.65	1992 ± 48.81
	**IL-8 (pg/ml)**		**IFNγ (pg/ml)**			
	**Chronic**	**Acute**	**Chronic**	**Acute**		
**D0**	72.08 ± 0.98	47.54 ± 2.53	U.D.	U.D.		
**D1**	855.4 ± 50.91	39.32 ± 2.08	U.D.	U.D.		
**D2**	673.7 ± 15.56	18.21 ± 1.16	U.D.	U.D.		
**D3**	196.3 ± 3.16	87.64 ± 1.61	U.D.	U.D.		
**D4**	36.74 ± 1.72	29.62 ± 1.58	U.D.	U.D.		
**D5**	35.23 ± 1.13	18.39 ± 1.47	U.D.	U.D.		
**D6**	40.64 ± 1.56	55.81 ± 1.54	U.D.	U.D.		
**D7**	20.19 ± 1.41	2361 ± 140.8	U.D.	39.79 ± 4.55		
**D8**	23.64 ± 0.37	2.48 ± 2.60	U.D.	U.D.		

### Statistical analyses

The student’s *t*-test was used for comparisons of two samples. P values < 0.05 were considered significant. Error bars indicate standard deviation (SD). The number of biological replicates is 2 and experimental replicates is 3, unless otherwise mentioned in Figure Legends.

## Results

### Determination of LPS-induced systemic inflammation

**Fig 1:**
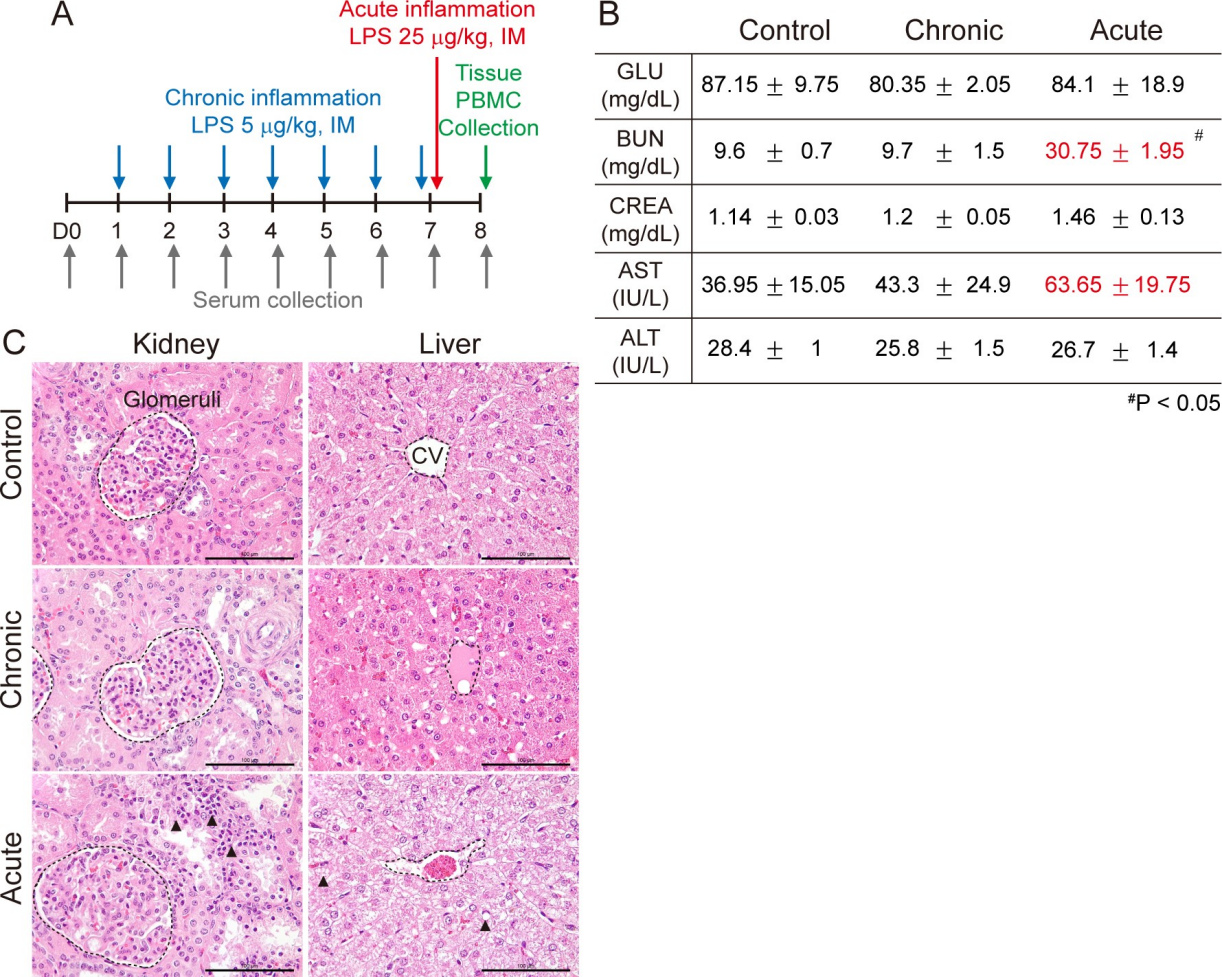
Comparison of serum chemistry and histopathology. **(A**) Experimental schematic: For chronic inflammation, 5 μg/kg lipopolysaccharide (LPS) was administrated, intramuscularly (IM), seven times, daily. For acute inflammation, 25 μg/kg LPS was administrated, IM, once at day7. Two CMS minipigs were used for each group (control, chronic, acute). Blood and tissue were sampled for further study. **(B)** Serum chemistry was measured using a TBA 120 FR chemistry analyzer (Toshiba Co., Japan). Absolute values are indicated. #*P*<0.05. **(C)** H&E staining. Glomeruli in kidney and central vein in liver are indicated with a black dot line. Abnormal lesions are indicated with black arrow head. Scale bar = 100 μm.

To develop the systemic inflammatory minipig model, seven doses of 5 μg/kg LPS were administrated for chronic inflammation, and one dose of 25 μg/kg LPS was administrated for acute inflammation (Fig 1A). To determine whether LPS induces organ damage, analyses of serum chemistry and histopathology were performed. LPS increased blood urea nitrogen (BUN) and aspartate aminotransferase (AST) in acute inflammation at day 8 (Fig 1B), indicating kidney and liver damage. H&E staining showed lymphoid infiltration in renal cortical interstitial, without glomeruli abnormality, and hydropic change of hepatocytes in acute inflammation (black arrowhead) (Fig 1C). There were no changes in serum chemistry and histopathology in chronic inflammation. These results show that LPS-induced systemic acute inflammation injures the kidney and liver, which is diagnosed by serum chemistry and histopathology.

**Fig 2:**
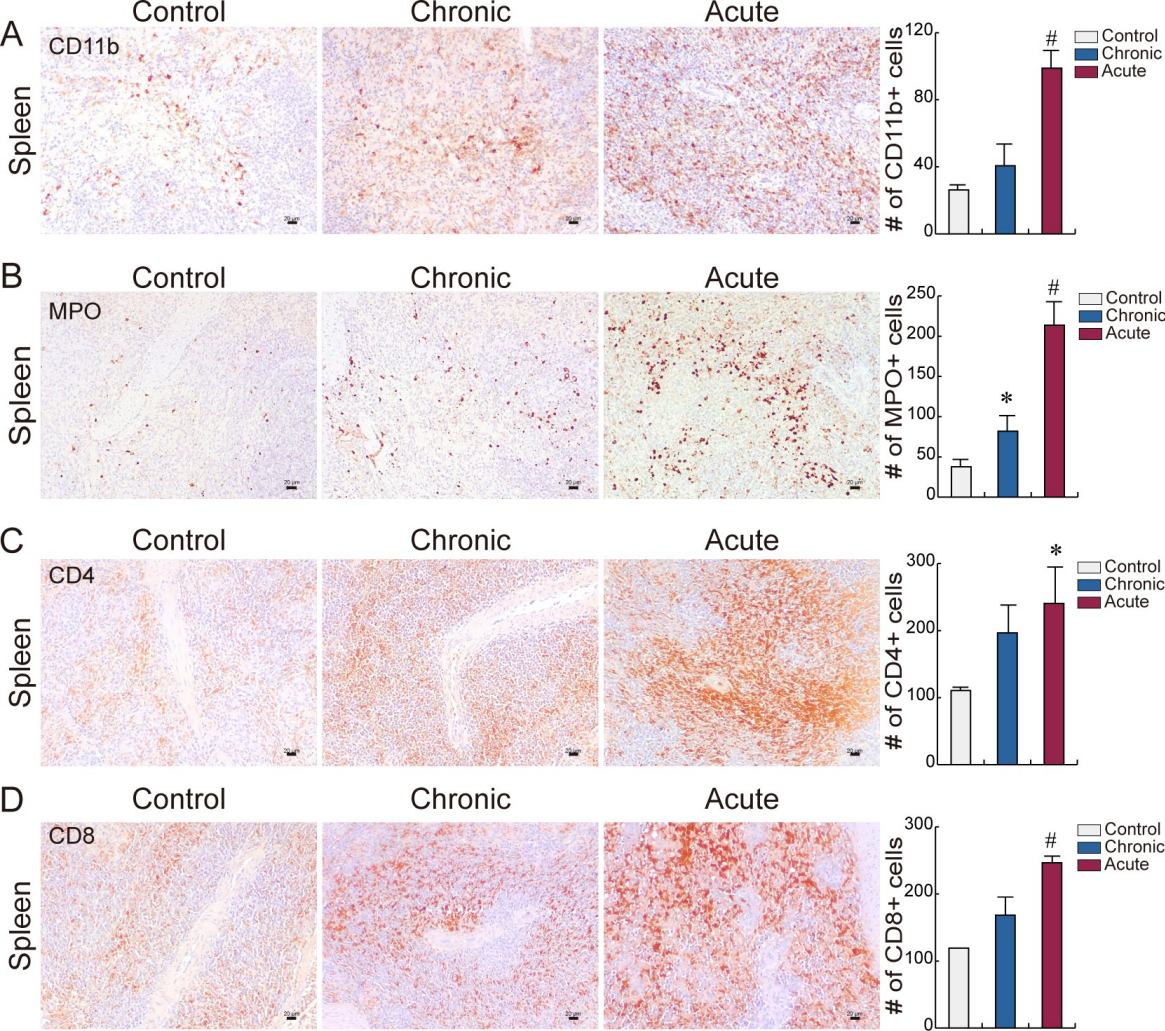
Immune cell distribution in spleen. **(A–D)** Immunohistochemistry. Population and distribution of **(A)** CD11b+ (macrophage), **(B)** MPO+ (neutrophil), **(C)** CD4+, and **(D)** CD8+ (lymphocyte) cells were examined in spleen. Representative images are shown; N≥3. Quantification of the immune cell number is shown as mean ± SD; ^#^*P*<0.01 relative to control, *P<0.05 relative to control. Scale bar = 20 μm.

The spleen filters and stores blood, therefore, the population of circulating immune cells can be measured in this organ. Thus, the IHC of immune cells following LPS induction was determined. The number of CD11b+, MPO+, CD4+, and CD8+ cells was slightly increased in chronic inflammation, whereas it massively increased in acute inflammation (Fig 2A–2D). These results indicate that IHC accurately detects the type and distribution of various immune cells. LPS induction establishes successful systemic inflammatory minipig model, and the direct detection of immune cells and serum chemistry compensate for inflammation diagnosis.

### Immune cell dynamics in LPS-induced pulmonary inflammation

**Fig 3:**
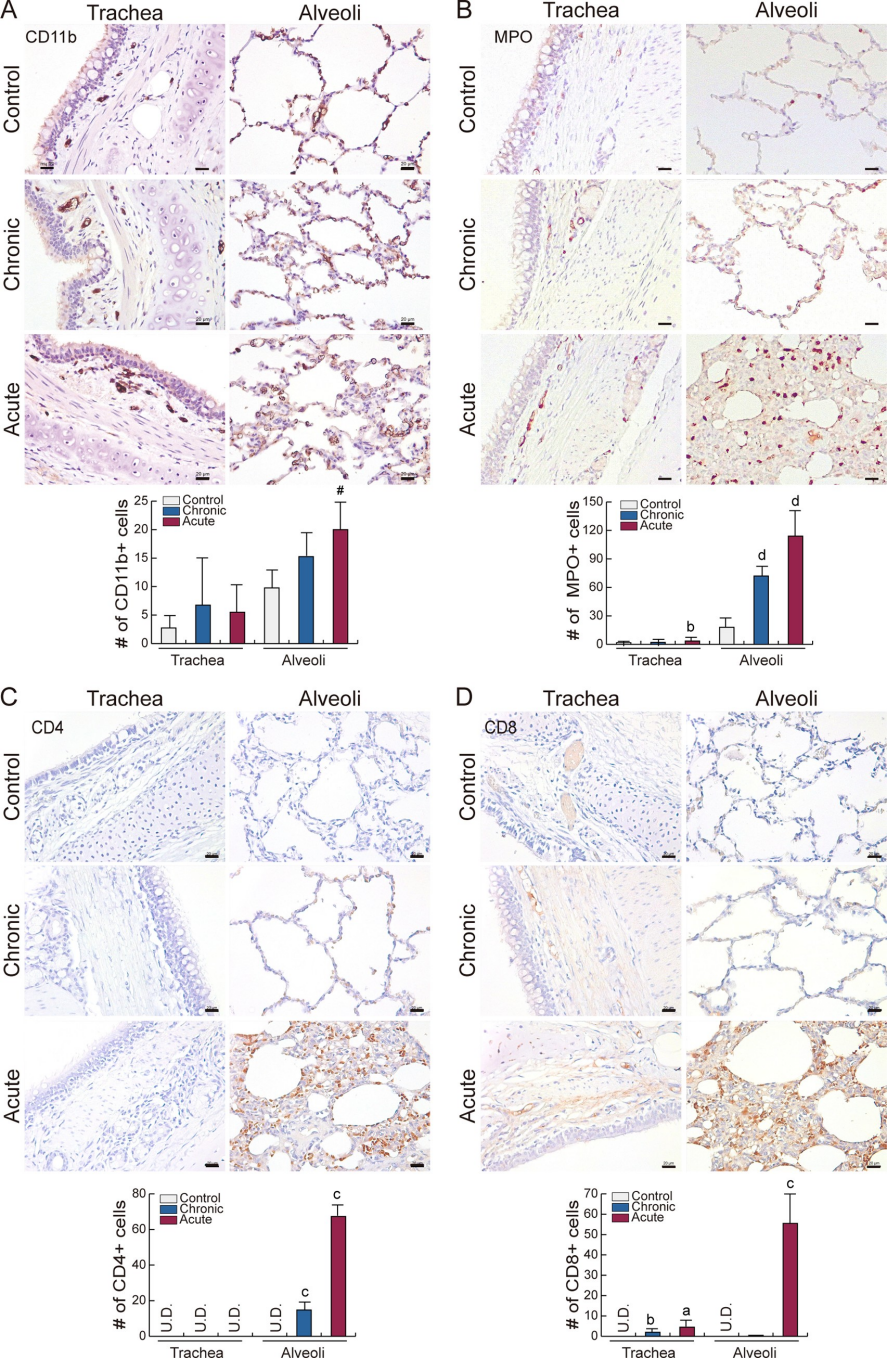
Immune cell distribution in lung. **(A–D)** Immunohistochemistry. Population and distribution of **(A)** CD11b+ (macrophage), **(B)** MPO+ (neutrophil), **(C)** CD4+, and **(D)** CD8+ (lymphocyte) cells were examined in lung. Representative images are shown; N≥3. Quantification of the immune cell number is shown as mean ± SD; ^a^*P*<0.01 relative to trachea control, ^b^*P*<0.05 relative to trachea control, ^c^*P*<0.01 relative to alveoli control, ^d^*P*<0.05 relative to alveoli control. Scale bar = 20 μm.

As the respiratory tract is directly connected with the outside of the body, alveolar macrophage is activated to remove foreign bodies in lung [20]. The function of other immune cells during homeostasis or inflammation are less well known. Thus, macrophages, neutrophils, and lymphocytes in the lung were detected after LPS induction using IHC. CD11b+ and MPO+ cells were identified, while CD4+ and CD8+ cells were not found in normal lung. All four types of immune cell were elevated in the alveoli during acute inflammation (Fig 3A–3D). Especially, CD4+ and CD8+ T lymphocytes significantly infiltrated alveoli after LPS induction. These results suggest that systemic inflammation regulates immune cell dynamics and infiltration into the damaged lung.

### Inflammation-related gene expression in tissue and PBMC

**Fig 4:**
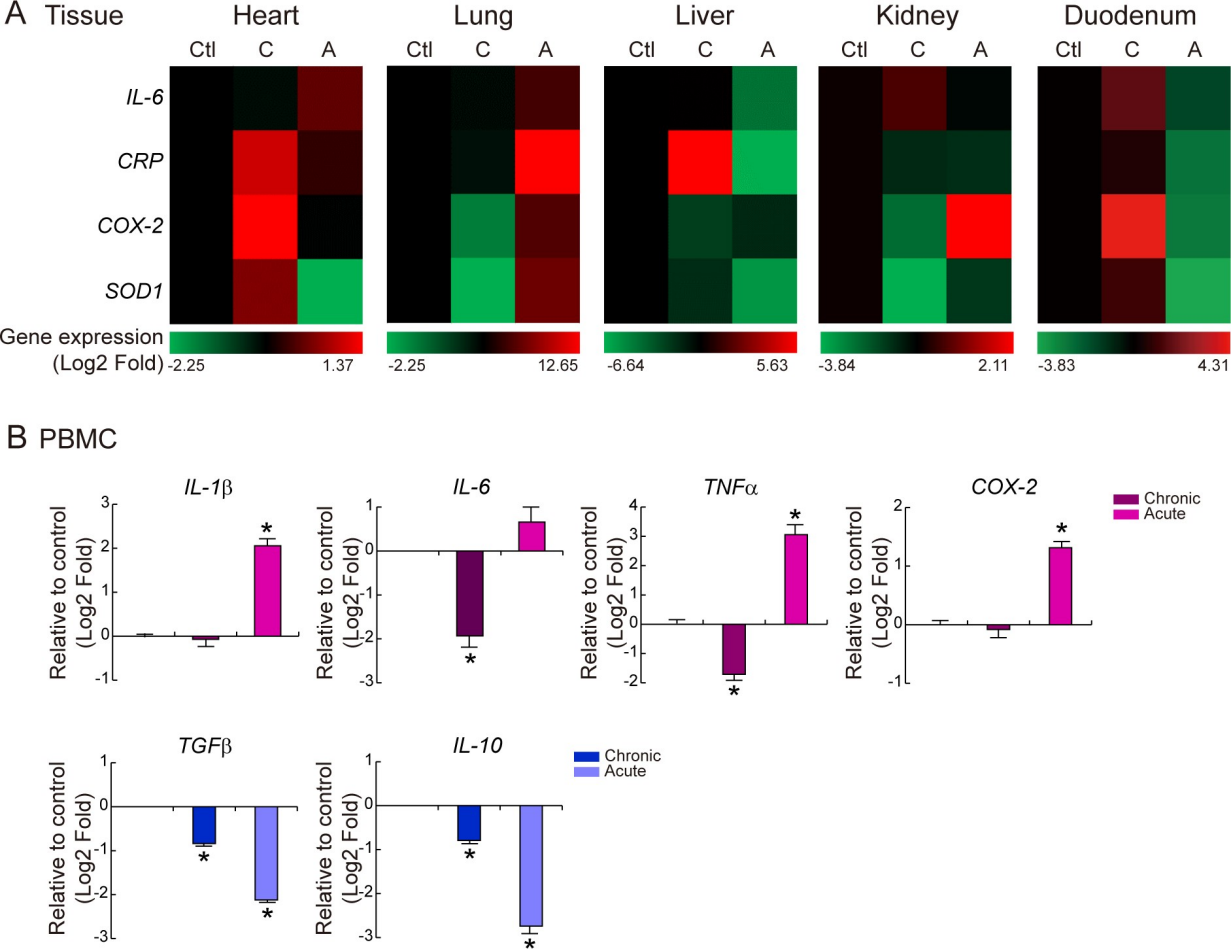
Inflammation-related gene expression in tissues and PBMC. **(A**) qRT-PCR and Heatmap in tissues. *IL-6*, *CRP*, *COX-2*, *SOD1* mRNA expression were analyzed in heart, lung, liver, kidney, and duodenum under chronic and acute inflammation with two biological and three experimental replicates. Red color indicates gene upregulation and green color indicates gene downregulation. **(B)** qRT-PCR in PBMC. Pro-inflammatory (*IL-1β*, *IL-6*, *TNFα*, *COX-2*) and anti-inflammatory (*TGFβ*, *IL-10*) mRNA expression were analyzed in PBMC under chronic and acute inflammation with two biological and three experimental replicates.

TLR4 has been identified in parenchymal organs including heart [21], lung [22], liver [23], kidney [24], and intestine [25], which suggests that LPS might directly affect these organs. LPS-bound TLR4 upregulates transcription factor NF-kB and numerous target gene synthesis, subsequently. Thus, to determine the effect of LPS on NF-kB target gene expression [26–29], the expression of cytokine (*IL-6*), acute phase inflammation response (*CRP*), pro-inflammatory (*COX-2*), and anti-oxidant (*SOD1*) was measured. Chronic cardiac inflammation upregulated *CRP* (1.1 fold change), *COX-2* (1.37), and *SOD1* (0.69), while acute upregulated *IL-6* (0.52). Chronic pulmonary inflammation decreased the *COX-2* (-1.61) and *SOD1* (-2.26) mRNA expression, while acute increased *IL-6* (3.29), *CRP* (12.64), *COX-2* (4.00), and *SOD1* (5.39) mRNA expression. Only *CRP* (5.62) was upregulated during chronic hepatic inflammation. The expression of *COX-2* (2.11) was increased in acute inflammation of kidney. In the intestine, LPS upregulated all four genes during chronic inflammation [*IL-6* (1.64), *CRP* (0.57), *COX-2* (4.30), and *SOD1* (1.06); Fig 4A]. These data suggest that each organ has different sensitivity to LPS (lung and kidney: immediate response; heart, liver, duodenum: delayed response) and each organ shows specific target gene upregulation.

To determine the synthesis of biomolecules in immune cells, PBMC was isolated and cytokine mRNA expression was analyzed. In chronic inflammation, both pro-inflammatory and anti-inflammatory genes were suppressed. In acute inflammation, pro-inflammatory genes (*IL-1β*, *IL-6*, *TNFα*, *COX-2*) were upregulated, while anti-inflammatory genes (*TGFβ*, *IL-10*) were downregulated (Fig 4B). PBMC which is stimulated by multiple low dose LPS downregulated the cytokine synthesis, suggesting attenuation of the immune response. In addition, single high dose LPS enhanced the pro-inflammatory cytokine synthesis, suggesting activation of the immune response.

### Absolute cytokine level after LPS-induced systemic inflammation

**Fig 5:**
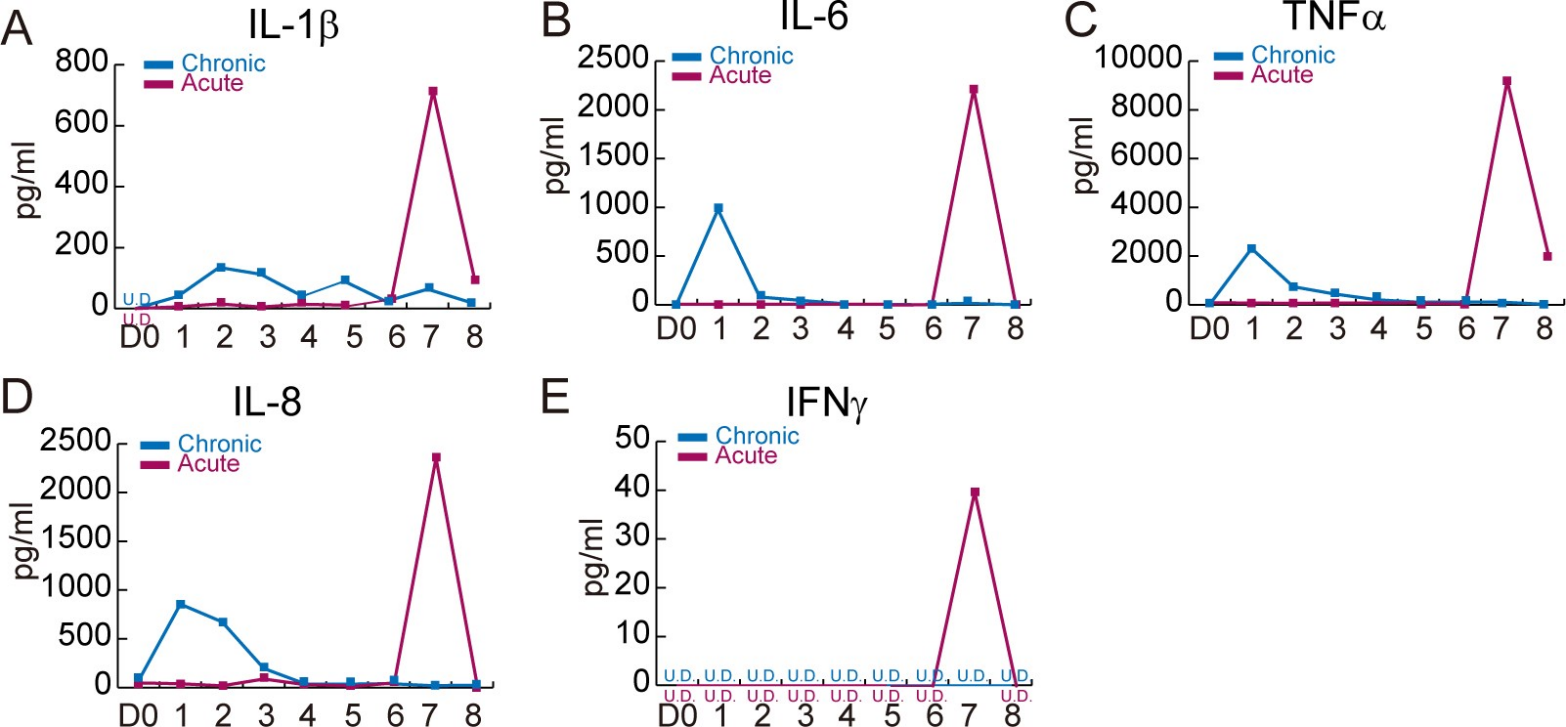
Serum cytokine analysis using ELISA. Blood was collected at day 0, 1, 2, 3, 4, 5, 6, 7, and 8 and then serum was isolated. Absolute cytokine levels (pg/ml) were measured by ELISA with two biological and three experimental replicates. **(A)** IL-1β, **(B)** IL-6, **(C)** TNFα, **(D)** IL-8, **(E)** IFNγ. U.D. = undetected.

To determine the inflammatory cytokine response after LPS induction, cytokines were detected in serum at various time points using ELISA. In chronic inflammation, the peak concentration of IL-6, TNFα, and IL-8 was two hours after the first LPS administration (5 μg/kg) and then cytokine levels gradually decreased despite daily LPS administration (Fig 5B–5D, Table 2). Moreover, IL-1β expression showed a delay in peak concentration after the second LPS challenge, and cytokine level was fluctuated until day 8 (Fig 5A, Table 2). IFNγ was not detected (Fig 5E, Table 2). In acute inflammation, all five cytokines reached their peak level two hours after LPS administration (25 μg/kg) (Fig 5A–5E, Table 2). The basal level of cytokines showed the variation in homeostasis (Fig 5, IL-1β: undetected, IL-6: 0.797 pg/ml, TNFα: 27.78 pg/ml, IL-8: 72.08 pg/ml, IFNγ: undetected). All five cytokines were upregulated by LPS both chronic and acute. However, the elevated level of cytokine was much higher in acute LPS induction compared to day 0. Interestingly, the cytokine level at day 8 returned to similar level as day 0.

## Discussion

In this study, inflammation-related gene expression in organs (heart, lung, liver, kidney, intestine), cytokine mRNA in PBMC, absolute cytokine in serum, and immune cell population in tissue were analyzed after LPS induction in CMS minipig. Rodent models are attractive inflammatory model due to cost effectiveness but their body size limits hemodynamic monitoring [14, 30, 31]. LPS modulation on the immune response is well characterized in NHP because of the close phylogenic relationship [32, 33]. In this study, the CMS minipig was used as a systemic inflammatory animal model. Porcine have different pulmonary vascular physiology to humans in systemic inflammation [31]. However, its high similarity in immune genes suggest that porcine can be another representative inflammatory animal model. The immune response is an effective process involving immune cells and cytokines. Acute inflammation increased the number of immune cells and distribution of all four immune cells (CD11b+ macrophage, MPO+ neutrophil, CD4+ lymphocyte, CD8+ lymphocyte) (Fig 2A–2D). Also, mRNA levels of pro-inflammatory cytokines in PBMC was upregulated (Fig 4B). Furthermore, cytokine level in serum was elevated after LPS induction (Fig 5). These results shows that LPS regulates the immune response, including immune cell dynamics, cytokine synthesis in immune cells, and elevation of cytokine in serum. 5 μg/kg LPS, seven times, IM administration induces mild and prolonged immune response indicating slight increase of immune cell population and serum cytokine level. Also, 25 μg/kg LPS, once, IM administration induces strong immune cell response indicating massive increase of immune cell population and serum cytokine level. Thus, in this study, a stable and affordable inflammatory minipig model and analytic tools for inflammation has been established.

Bacterial LPS mediates the sepsis and immune response, which leads to tissue damage and organ failure [34]. Especially, LPS-treated liver and kidney failure have been reported, which were diagnosed by serum chemistry [35, 36]. As AST is located in the cytosol and mitochondria of hepatocytes, and alanine aminotransferase (ALT) is situated in the cytosol of hepatocyte, elevation of AST or ALT in serum is the most relevant marker of hepatic injury. BUN and creatinine levels are also elevated when renal clearance fails. In this study, we have found that LPS-induced BUN and AST elevation (Fig 1B) and *COX-2* upregulation in acute renal inflammation and *CRP* upregulation in chronic hepatic inflammation (Fig 4A). Previous studies support our findings. Enhanced COX-2 is considered a renal pathological condition as COX-2 regulates tubular reabsorption and glomeruli filtration [37–39]. As CRP is synthesized in hepatocytes, elevated CRP is related to hepatic damage [40]. These results show that LPS mediates organ damage and organ-specific gene upregulation under inflammation. Thus, organ-specific target genes can be analytic tools for inflammation.

Pulmonary inflammation was significantly affected by acute inflammation, as demonstrated by immune cell infiltration and NF-kB target gene (*IL-6*, *CRP*, *COX-2*, *SOD1*) upregulation (Figs 3 and 4A). Moreover, those genes were downregulated in chronic inflammation even though there was a slight increase of immune cell infiltration. This could be because activated neutrophil or macrophage regenerate the lung and attenuate the inflammation [41, 42]. Of note, NF-kB target genes were upregulated in heart and intestine during chronic inflammation (Fig 4A) which indicates that these organs are affected by the long-term immune response [43, 44]. These findings suggest that each organ has distinct LPS sensitivity (acute or chronic), and LPS-induced tissue damage has various mechanisms (specific target). It is noteworthy examining a serum chemistry and a histopathology to determine the organ damage. However, it does not completely reflect the various disease conditions, such as organ specific damage and injury/recovery status. In this study, we have developed LPS-induced systemic acute or chronic inflammation minipig model proven by immune cell population, cytokine mRNA expression in PBMC, and cytokine level in serum. Also, we have found that organ specific gene activation with various mechanism under systemic inflammation.

## Conclusions

LPS-mediated systemic inflammation affects the organs and novel analytic tools such as IHC of immune cells, cytokine mRNA analysis in PBMC, and absolute cytokine analysis in serum support conventional inflammation detection tools.
